# The Role of Malic Enzyme on Promoting Total Lipid and Fatty Acid Production in *Phaeodactylum tricornutum*

**DOI:** 10.3389/fpls.2018.00826

**Published:** 2018-06-19

**Authors:** Bao-Hua Zhu, Rui-Hao Zhang, Na-Na Lv, Guan-Pin Yang, Yi-Sheng Wang, Ke-Hou Pan

**Affiliations:** ^1^Key Laboratory of Mariculture, Ministry of Education, Ocean University of China, Qingdao, China; ^2^College of Marine Life Sciences, Ocean University of China, Qingdao, China; ^3^Qingdao National Laboratory for Marine Science and Technology, Qingdao, China

**Keywords:** malic enzyme, lipid, fatty acid, NADPH, *Phaeodactylum tricornutum*

## Abstract

To verify the function of malic enzyme (ME1), the ME1 gene was endogenously overexpressed in *Phaeodactylum tricornutum*. Overexpression of *ME1* increased neutral and total lipid content and significantly increased saturated fatty acids (SFAs) and polyunsaturated fatty acids (PUFAs) in transformants, which varied between 23.19 and 25.32% in SFAs and between 49.02 and 54.04% in PUFAs, respectively. Additionally, increased ME1 activity was accompanied by elevated NADPH content in all three transformants, indicating that increased ME1 activity produced additional NADPH comparing with that of WT. These results indicated that ME1 activity is NADP-dependent and plays an important role in the NADPH levels required for lipid synthesis and fatty acid desaturation in *P. tricornutum*. Furthermore, our findings suggested that overexpression of endogenous *ME1* represents a valid method for boosting neutral-lipid yield in diatom.

## Introduction

Malic enzyme (ME) is widespread in all kinds of organisms and catalyzes the reversible oxidative decarboxylation of malate to pyruvate, CO_2,_ and NAD(P)H in the presence of a divalent metal ion (Drincovich et al., 2001). Malic enzymes are divided into three categories on the basis of the substrate specificity and coenzyme preference: NAD**^+^**(EC 1.1.1.38-39), NADP**^+^**-dependent (EC1.1.1.40), or dependent upon both cofactors. These enzymes localize to the cytoplasm, mitochondria or chloroplasts in eukaryotes (Tang et al., 2010); however, little is known about the molecular basis for cofactor selectivity of these enzymes (Chang and Tong, 2003).

In spite of decades of research, the physiological function of ME remains poorly understood, and its role might vary from organism to organism (Kendrick and Ratledge, 1992). ME plays a crucial part in the provision of NADPH to promote desaturation and elongation reactions leading to the formation of polyunsaturated fatty acids (PUFAs) in some oleaginous organisms (Kendrick and Ratledge, 1992; Ren et al., 2013; Ratledge, 2014; Lv et al., 2016). Previous studies also reported novel physiological functions associated with this enzyme, including renovating UV-induced damage in maize seedlings (Drincovich et al., 1998; Casati et al., 1998), affecting the development of chloroplasts by generating excessive reducing power in transgenic C3 plants (Takeuchi et al., 2000), lengthening the lifespan of *Drosophila* during the larval stage (Kim et al., 2015), keeping stable levels of TCA-cycle intermediates in the bacterium *Sinorhizobium meliloti* (Zhang et al., 2016), and acting as a potential target of cancer chemotherapy (Chang and Tong, 2003) and as an anaplerotic enzyme in *Saccharomyces cerevisiae* (Zelle et al., 2011) and *Streptomyces coelicolor* (Rodriguez et al., 2012).

Few studies have centered on the role of MEs in lipid accumulation, with those undertaken focusing mainly in plants and mammals; however, little is known about the role of these enzymes in microalgae. An NADP-dependent ME was proposed for diatoms, with possible function as a decarboxylase of releasing CO_2_ in chloroplasts of diatoms, similar to that in C4 plants (Granum et al., 2005). The gene encoding ME in *Dunaliella parva* was cloned and characterized, but further research on the function of this gene was not conducted (Shang et al., 2012). Kroth et al. (2008) reported that *Phaeodactylum tricornutum* appears to contain two mitochondrial ME that are either NAD- or NADP-dependent. One of the ME-encoding genes was overexpressed endogenously in *P. tricornutum* (PtME), revealing its predominant localization to the mitochondria, as well as its significant impact on promoting lipid accumulation; however no further studies were performed on the role of ME in increasing lipid content (Xue et al., 2015).

The marine diatom *P. tricornutum* represents a potential producer of biodiesel because of its rapid growth, lipid-accumulation capability, and the availability of genetic tools (Zaslavskaia et al., 2000). Therefore, it is possible to genetically manipulate the key genes involved in fatty acid synthesis in this alga to enhance characters to gain both high lipid and high biomass levels necessary for industrial production.

To verify ME function, the ME1 gene from *P. tricornutum* (different from that studied by Xue et al., 2015), which possesses a mitochondria presequence and might have a dinucleotide-binding site given for NADP (Kroth et al., 2008), was overexpressed in *Escherichia coli* (Lv et al., 2016) and in *P. tricornutum* (this study). The results of this study, as well as those previously published (Lv et al., 2016), indicated that ME1 from *P. tricornutum* is NADP-dependent and can supply enough NADPH for both fatty acid biosynthesis and desaturation in *E. coli* (Lv et al., 2016) and *P. tricornutum* (this study).

## Materials and Methods

### Strains and the Growth Conditions

Wild-type (WT) *P. tricornutum* Bohlin (LAMB118), provided by Institute of Hydrobiology, Chinese Academy of Sciences, and zeocin-resistant colonies (named as PtME1-1, PtME1-2, and PtME1-3) were cultured in f/2 medium (Guillard, 1975) prepared with sterile seawater at 20 ± 1°C and under 37.50 μmol photons m^-2^ s^-1^, following a 12:12 photoperiod. Three cultures for every transformant and the WT strain (300-mL each) were cultivated axenically to monitor their growth. To set up growth curves, the optical density (OD) was measured at 750 nm every 2 days using a UV-3310 spectrophotometer (Hitachi, Tokyo, Japan) (Griffiths et al., 2011).

### Construction of the Plasmid Containing the ME1 Gene and Particle Bombardment Transformation

The *ME1* gene was amplified by polymerase chain reaction (PCR) using cDNA from *P. tricornutum* (GenBank accession: XP_002177890.1) as the template and the primers PtME1-F (GGGGTACCATGATATCATCGGCGTGTCG) and PtME1-R (GCTCTAGACTAGTGGTGGTGGTGGTGGTGGATTGATATTTCTCGTTTTTCC). To generate the pPha-T1-ME1 recombinant plasmid, the amplified gene was inserted into the pPha-T1 (Zaslavskaia et al., 2000) plasmid using an *fcp*A promoter driving the *ME1* gene, and the resistant strains were selected with Zeocin^TM^ (Invitrogen, Carlsbad, CA, United States).

The recombinant plasmid (pPha-T1-ME1) was introduced into *P. tricornutum* by a Bio-Rad Biolistic PDS-1000/He particle-delivery system (Bio-Rad, Hercules, CA, United States), according to the methods described by Zaslavskaia et al. (2000) and Zhu et al. (2016). It should be pointed out that seawater f/2 medium was used for culturing in this study.

### Genomic DNA Extraction and Molecular Identification

Total genomic DNA was isolated in the light of the method described by Watanabe et al. (1998). Transformants were screened by PCR using the gene-specific *Sh-ble* and *PtME1-ble* primers (**Table 1**), respectively. To prepare a digoxigenin-labeled probe for Southern blot analysis (Falciatore et al., 1999), the *ble* fragment was used as the template and genomic DNA was digested with *EcoR* I and *Kpn*I, respectively. For western blot (Poulsen and Kröger, 2005), an anti-His-tag antibody (Bioss, Woburn, MA, United States) was used to detect the ME1 protein, and actin was served as the internal control. Cells were harvested by centrifugation at late exponential stage for assays.

**Table 1:** Primers used in this study.

Primer name	Primer sequence	(5′-3′)
*PtME1*-FGGGGTACCATGATATCATCGGCGTGTCG		
*PtME1*-RGCTCTAGACTAGTGGTGGTGGTGGTGGTGGATTGATATTTCTCGC		
TTTTTCC		
*Sh ble*-FCCAACAGCATCACCCAGAT		
*Sh ble*-RGGTAGAACTCGTCGCTCAGG		
*ME1- ble*-FGGGCTGGGAGCATCAGTTTG		
*ME1-ble*-RACCCAGGCCAGGGTGTTGTC		
RT-PtME1-F	GTGTCGTGGCAGCCTGAAATC	
RT-PtME1-R	CGGACCGAAATCCTTATTGGTATCA	
RT-H4-F	GTGGTAAAGGAGGCAAGGGTC	
RT-H4-R	CACGGGTCTCTTCGTAAATC	


*Underlined bases and double underlines indicate the nucleotide sequences recognized by and digested by restriction endonucleases and encoding the His tags, respectively.*

### RNA Extraction and Quantitative Real-Time (qPCR)

RNA extraction and qPCR were performed as previously described (Zhu et al., 2016) using primers shown in **Table 1**. The histone H4 gene was served as the internal reference (Siaut et al., 2007). The 2^-ΔΔCt^ method (Livak and Schmittgen, 2001) was used to analyze the *ME1* expression and calculate relative *ME1-*transcript abundance.

### Analytical Methods for Measuring Total and Neutral-Lipid Contents

Total lipids were extracted according to the method as described previously (Zhu et al., 2016). To detect cellular neutral-lipid content in *P. tricornutum*, BODIPY505/515 (Invitrogen) staining was carried out according to the protocol described by Cooper et al. (2010). Algal cells (10^6^ cells/mL) were first treated with 2% dimethyl sulfoxide (DMSO) for 10 min at room temperature. A stock solution of 100 μg/mL BODIPY505/515 was prepared using anhydrous DMSO and added directly to algal solution to obtain a final BODIPY 505/515-labeling concentration of 0.87 μg/mL, followed by incubation in darkness for 10 min at room temperature. Stained cells were detected by their fluorescence intensity with flow cytometry (BD FACSVantage SE; BD Biosciences, Franklin Lakes, NJ, United States). Excitation and emission wavelengths were 485 nm and 535 nm, respectively. Quantitative comparison of neutral-lipid content between samples was obtained according to relative fluorescence-intensity values.

### Determination of Fatty Acid Composition

Fatty acid methyl esters were analyzed using gas chromatography (Agilent 6890 Series GC System; US10251016; Agilent, Santa Clara, CA, United States) as previously described (Zhu et al., 2014) after transmethylation according to a method described by Lepage and Roy (1984). Mixed external standards of fatty acids (Supelco 37, United States) were used to detect and determine the Fatty acid composition.

### Measurement of ME Enzyme Activity

Malic enzyme activity in *P. tricornutum* was measured using an NADPH-ME kit (Solarbio, Beijing, China) according to manufacturer instructions. The optimum reaction system was prepared as followed: 50 mM, pH 7.5 Tris–HCl, 1 mM MgCl_2_, 0.5 mM NADP^+^, 10 mM L-malate. Soluble protein concentration was quantified using a Bradford assay kit (Genmed Scientifics, Shanghai, China). ME activity was determined by monitoring the change in absorbance at 1-min intervals continuously at 340 nm using a UV-3310 spectrophotometer (Hitachi). One unit of ME activity was defined as 1 μM NADPH generated by 1 mg protein per minute in the reaction system:

ME ⁢(U / mg protein)=[(A2−A1)/6.22]×(1/t)×(V1/V2)/C

where A1 is the initial absorbance, A2 is the absorbance after the reaction, 6.22 represents the extinction coefficient per mM NADPH, t is the reaction time (1 min), l is the path length of the cuvette (1 cm), V1 is the total reaction volume (900 μL), V2 is the volume of ME solution (30 μL), and C is the concentration of protein (mg/mL).

### NADPH-Content Analysis

NADPH content in *P. tricornutum* was analyzed with the Amplite Fluorimetric NADP/NADPH ratio assay kit (AAT Bioquest, Sunnyvale, CA, United States) according to manufacturer instructions. After lysis and ultrasonication extraction, microalgal samples were centrifuged at 8000 × *g* for 10 min at 4°C, and the supernatant was prepared for testing. Traditional NADPH assays are performed by monitoring the changes in NADPH absorption at 340 nm using a Synergy microplate reader (BioTek, Winooski, VT, United States). The excitation and emission wavelengths used were 540 nm and 590 nm, respectively. The concentration of NADPH was determined from the standard curve of NADPH.

### Statistical Analysis

One-way analysis of variance was used at a level of significance of *P* < 0.05 to calculate significant differences between treatments using SPSS 17.0 software (IBM, Armonk, NY, United States), and all data are reported as the mean ± standard deviation (three replicates were used, *n* = 3).

## Results and Discussion

### Effect of the Transgene on *P. tricornutum* Growth

**FIGURE 1:**
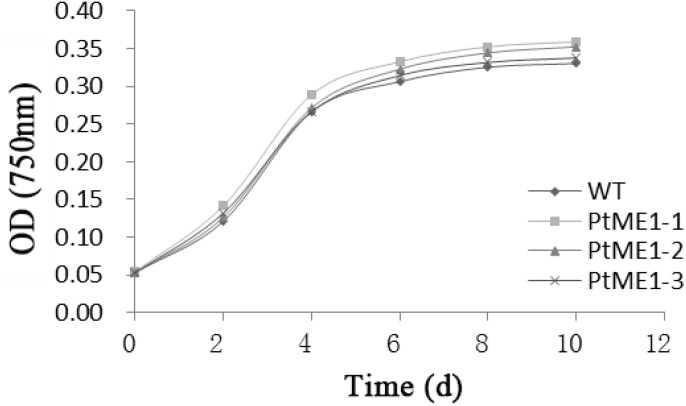
Growth curves of transgenic clones and the WT strain. Values represent averages of three replicates (*n* = 3).

Similar growth curves were shown in **Figure 1** for both the WT strain and transformants although all the resistant strains showed a slightly increased growth rate and did not differ significantly from that of the WT strain, which suggested that the transgene exerted a temperate influence on the growth of the transformants. In contrast to this study, Jiang et al. (2013) reported that depletion of *ME1* or *ME2* strongly impaired tumor-cell growth, and overexpression of these genes enhanced tumor-cell growth. By contrast, growth reductions in transgenic strains were reported by other studies (Li et al., 2010; Radakovits et al., 2011). Therefore, further research is needed to understand the underlying mechanism.

### Transgene Detection via Molecular Approaches

**FIGURE 2:**
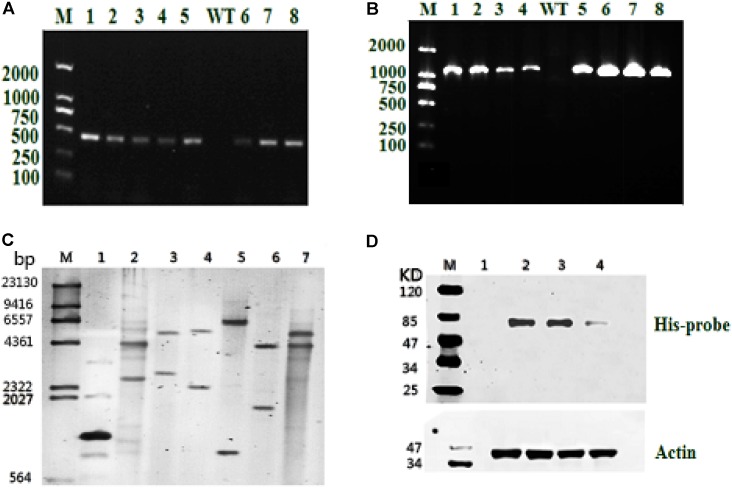
Molecular analysis of resistant clones. **(A)** PCR of the resistant gene *Sh-ble* in selected clones and the WT strain. M: DNA-size marker. **(B)** PCR performed using primers designed from tandem *ME1* and *ble* sequences in the recombinant plasmid pPha-T1-ME1. The reference band is 1126 bp. M: DNA-size marker. **(C)** Southern blot analysis of *EcoR* I-digested products (lane 1: positive control represents the plasmid, pPha-T1; lanes 2–4: PtME1-1, PtME1-2, and PtME1-3) and *Kpn* I-digested products (lanes 5–7: PtME1-1, PtME1-2, and PtME1-3) from genomic DNA with a *ble* fragment used as a digoxigenin-labeled probe. **(D)** Western blot analysis using an anti-His-tag antibody. Actin was used as an internal control (lanes 2–4: PtME1-1, PtME1-2, and PtME1-3).

Resistant clones were first selected by growth on f/2 solid medium (1% agar) with 100 μg/mL Zeocin. Further screening was conducted by PCR using the gene-specific *Sh-ble* and *ME1-ble* primers (**Table 1**). As shown in **Figures 2A,B**, all resistant clones presented the expected fragment sizes, but no DNA band was seen in the WT strain. To further verify gene integration, Southern blot analysis was performed in all three resistant strains and the WT strain. As shown in **Figure 2C**, all three resistant strains showed two or more hybridized bands detected with the *ble* probe digested using different restriction enzymes, indicating that exogenous *sh-ble* and endogenous *ME1* had been integrated into the *P. tricornutum* genome, and that these clones were undoubted transformants. To detect target protein levels translated from the introduced *ME1* gene in all the resistant strains, western blot was conducted with an anti-His-tag antibody. A cross-reacting band, the same size as expected, was shown in all the resistant strains, whereas this was not observed in the WT strain (**Figure 2D**).

**FIGURE 3:**
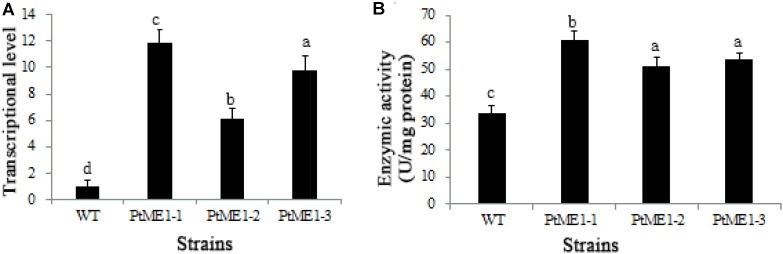
Molecular characterization of resistant clones. **(A)** Relative quantification of *ME1* transcripts in the WT strain and the three *Phaeodactylum tricornutum* resistant clones. *ME1-*transcript abundance in the resistant clones was quantified with WT cells as the standard and normalized to endogenous histone *H4* expression (*n* = 6). **(B)** NADP-ME activity (U/10^6^ cells) in the WT strain and the three *P. tricornutum* transformants. Activity assays were performed on samples from the same experiments. Values represent averages of at least three replicates. Error bars indicate standard deviations. Values with different letters (a,b,c,d) indicate a significant difference between them (*p* < 0.05).

The impact of *ME1* overexpression on *ME1* mRNA levels was investigated by qPCR analysis in three resistant clones and the WT strain. As shown in **Figure 3A**, all transformants displayed elevated *ME1*-transcript abundance as compared with the WT strain. Additionally, the relative *ME1-*transcript abundance of the transformants was 6.09–11.87 time higher than that of the WT strain. Furthermore, the increase in *ME1-*transcript abundance was accompanied by enhanced ME1 activity (**Figure 3B**), which increased 1.52–1.81 fold relative to that observed in the WT strain. These results validated these resistant colonies as the expected transformants.

### ME1 Overexpression Increases Neutral and Total Lipid Contents

**FIGURE 4:**
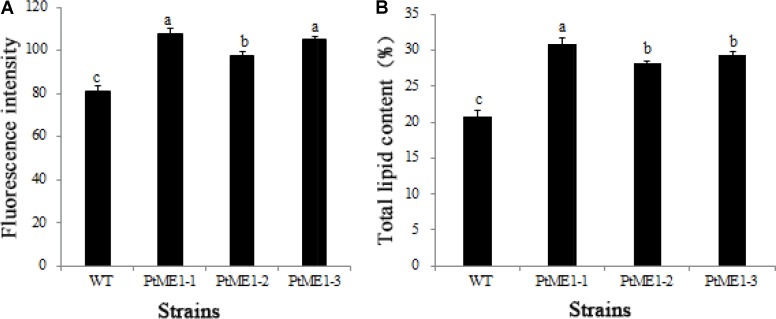
Neutral-lipid accumulation and total lipid contents. **(A)** Neutral-lipid and **(B)** total lipid contents in the WT strain and the three *Phaeodactylum tricornutum* transformants. Values represent averages of three replicates (*n* = 3). Values with different letters (a,b,c) indicate a significant difference between them (*p* < 0.05).

As shown in **Figure 4A**, the neutral-lipid content of all three transformants (PtME1-1, PtME1-2, and PtME1-3) increased significantly (*P* < 0.05; 33.33, 20.25, and 29.63%, respectively) as compared with that in the WT strain. Additionally, total lipid content of the three resistant clones was enhanced significantly, and that of the PtME1-1 transformant increased by 48.42% as compared with that of the WT strain (**Figure 4B**). These findings suggested that *PtME1* overexpression induced the accumulation of neutral- and total lipids in *P. tricornutum*. Similarly, overexpression of two exogenous *ME* genes in *M. circinelloides* led to a 2.5-fold increase in lipid accumulation (Zhang et al., 2007). Moreover, *PtME* expressed in the green microalga *Chlorella pyrenoidosa* resulted in a 3.2-fold increase in neutral-lipid content relative to that observed in the WT strain, with total lipid content reaching 40.9% (dry cell weight) (Xue et al., 2016).

A previous study reported that overexpressing *ME* in *E. coli* led to a 4-fold increase in intracellular lipids by providing a high level of NADPH (Meng et al., 2011). In a previous study with different methods (the recombinant plasmid pHY-PtME instead of pPha-T1-ME1, transformation by electroporation rather than Biolistic PDS-1000/He particle-delivery system) to the present study, *PtME* overexpressed endogenously in *P. tricornutum*, resulting in a marked increase (2.5-fold) in total lipid content in transgenic strains, reaching a 57.8% dry cell weight and a similar growth rate to that observed in the WT strain (Xue et al., 2015). However, in that study, the authors did not confirm the NADP^+^-dependence of the ME-encoding gene, suggesting that it would not necessarily have produced NADPH. Therefore, the mechanisms associated with the increases in lipids remains unclear.

### The Effects of ME1 Overexpression on Fatty Acid Composition

As shown in **Table 2**, Significant decreases in monounsaturated fatty acids (MUFAs) from 24.61 to 19.96% were detected in transgenic microalgae. With regard to saturated fatty acids (SFAs) and PUFAs, marked increases in the transgenic lines, from 23.19 to 25.32% and from 49.02 to 54.04%, respectively, were observed, revealing that overexpression of endogenous *ME1* facilitated SFA and PUFA biosynthesis. In contrast to the present study, other *PtME*s different from those in this study were overexpressed endogenously in *P. tricornutum*, resulting in an increase in MUFAs and a slight decrease in PUFAs in transgenic microalgae (Xue et al., 2015). Wynn and Ratledge (1997) reported that enhanced ME activity leads increases in the cytosolic NADPH pool, and that ME plays a vital role in the provision of NADPH for storage lipids synthesis by *Aspergillus nidulans*. These findings indicated that the function of ME in lipid biosynthesis involves supplying NADPH for fatty acid desaturation (Liang and Jiang, 2013).

**Table 2:** Fatty acid composition of the WT strain and the three transformants (% of total fatty acids).

	Strains				
	
Fatty acid	WT	PtMEl-1	RME1-2	PtMEl-3	Mean value
C14:0	8.31 ± 0.09^a^	8.62 ± 0.35^a^	9.26 ± 0.30^b^	8.60 ± 0.13^a^	8.83 ± 0.37^ab^
C16:0	12.10 ± 0.39^a^	13.13 ± 0.66^b^	13.04 ± 0.41^b^	12.91 ± 0.35^b^	13.03 ± 0.11^b^
C16:l	23.66 ± 0.52^c^	18.11 ± 0.70^ab^	17.72 ± 0.61^a^	19.16 ± 0.11^b^	18.33 ± 0.74^ab^
C16:2	1.62 ± 0.03^c^	1.52 ± 0.06^b^	1.50 ± 0.05^ab^	1.43 ± 0.03^a^	1.48 ± 0.05^ab^
C16:3	13.63 ± 0.60^a^	17.13 ± 0.91^c^	14.87 ± 0.41^ab^	15.09 ± 0.13^ab^	15.70 ± 1.25^b^
C18:0	2.77 ± 0.29^a^	3.02 ± 0.06^a^	2.89 ± 0.17^a^	3.71 ± 0.10^b^	3.21 ± 0.44^a^
C18:l	1.29 ± 0.05^a^	1.41 ± 0.06^a^	1.60 ± 0.35^ab^	1.86 ± 0.12^b^	1.62 ± 0.23^ab^
C18:2	3.09 ± 0.06^a^	3.24 ± 0.21^a^	3.16 ± 0.09^a^	3.20 ± 0.35^a^	3.20 ± 0.04^a^
C20:5	29.53 ± 0.46^a^	29.93 ± 0.20^ab^	30.48 ± 0.22^b^	29.73 ± 0.36^a^	30.05 ± 0.39^ab^
C22:6	1.15 ± 0.09^a^	2.22 ± 0.14^b^	2.66 ± 0.13^c^	2.76 ± 0.19^c^	2.54 ± 0.29^bc^
SFA	23.19 ± 0.15^a^	24.78 ± 0.87^b^	25.19 ± 0.45^b^	25.23 ± 0.49^b^	25.07 ± 0.25^b^
MUFA	24.61 ± 0.19^c^	19.52 ± 0.76^a^	19.33 ± 0.29^a^	21.02 ± 0.23^b^	19.96 ± 0.92^a^
PUFA	49.02 ± 0.80^a^	54.04 ± 0.70^c^	52.67 ± 0.29^b^	52.21 ± 0.59^b^	52.98 ± 0.95^bc^


*The values in the same row with different superscripts represent statistically significant differences (*P* < 0.05). Values represent averages of three replicates (*n* = 3).*

It is likely that the elevated ME activity generated NADPH for fatty acid desaturases and also increased fatty acid desaturase activity. (Zhang et al., 2007). The present study showed a 1.52- to 1.81-fold increase in ME1 activity (**Figure 3B**) as compared with that in the WT strain, which led to an increase in fatty acid desaturase activity and induced further accumulation of PUFAs in transgenic microalgae. A previous study showed that docosahexaenoic acid content increased significantly by adding ME based on the resulting elevation in ME activity and NADPH supply during a specific fermentation stage in *Schizochytrium s*p. HX-308 (Ren et al., 2009). Exogenously overexpressing *ME* in the green microalga *C. pyrenoidosa* results in a 34% increase in PUFAs in the transformed line, with the content of C16:3 and C18:3 lipids in the transgenic lines also elevated by 68.5 and 42.9%, respectively (Xue et al., 2016). Overexpressing *ME1* in *E. coli* showed that C14:0, C16:0, C18:1, and total fatty acid contents were increased by 34.8, 69.9, 54.2, and 50.2%, respectively, and that the content of C16:1 lipids was elevated 5.6-fold as compared with that of controls (Lv et al., 2016). These findings suggested that *PtME* influenced fatty acid composition by regulating lipogenesis (Xue et al., 2016).

### The Role of ME1 in NADPH Generation in *P. tricornutum*

**FIGURE 5:**
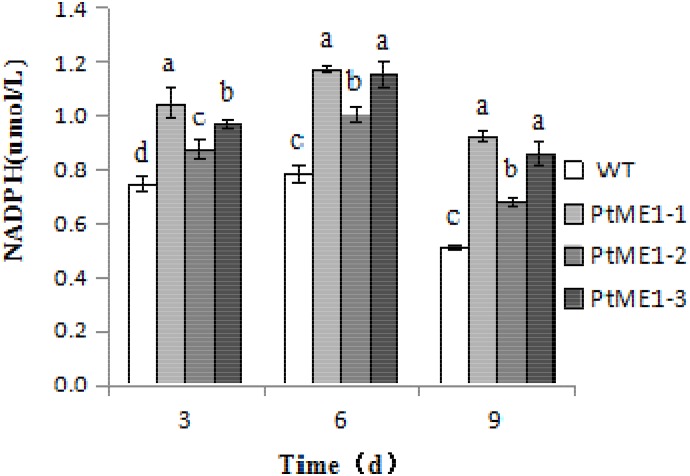
NADPH content in the WT strain and the three *Phaeodactylum tricornutum* transformants. Values represent averages of three replicates (*n* = 3). Values with different letters (a,b,c,d) indicate a significant difference between them (*p* < 0.05).

To verify whether *ME1* overexpression can promote NADPH production, NADPH content was measured in the three transformants and the WT strain during cultivation on days 3, 6, and 9. As shown in **Figure 5**, the NADPH content of all algal strains increased markedly on day 6 and decreased significantly on day 9, indicating that NADPH could be used to produce other compounds, such as lipids, and expended during the late exponential-growth phase. However, the NADPH content of three transformants was clearly enhanced relative to that observed in the WT strain during the cultivation stage, increasing by 40.6, 17.8, and 30.0% on day 3, 49.3, 28.4, and 46.7% on day 6, and 80.3, 33.2, and 68.2% on day 9 for PtME1-1, PtME1-2, and PtME1-3, respectively, suggesting that *ME1* overexpressing significantly improved NADPH biosynthesis in *P. tricornutum*. Furthermore, the increase in ME1 activity was accompanied by elevated NADPH content, indicating that increased ME1 activity produced additional NADPH.

NADPH plays a crucial part in fatty acid accumulation in oleaginous organisms, with these organisms exhibiting a unique mechanism involving NADPH supply. Wynn et al. (1999) reported that ME was important for providing sufficient NADPH for lipid biosynthesis in the fungi, hypothesizing that lipid production was controlled by ME-mediated NADPH generation to fatty acid synthase activity, given that no other enzyme activity has displayed such a strong link with the content of lipid accumulation. A previous report indicated that ME represents the only NADPH source for fatty acid biosynthesis in the yeast *Rhodosporidium toruloides* (Evans and Ratledge, 1985). Furthermore, *ME* overexpression in *E. coli* resulted in increased lipid accumulation in engineered strains relative to controls due to two potentially linked results: increased NADPH and pyruvate levels (Meng et al., 2011).

Until now, no study reported whether ME was capable of providing NADPH in eukaryotic microalgae. Xue et al. (2015) reported that *PtME* overexpression significantly impacted the promotion of lipid accumulation, attaining a 57.8% increase in dry cell weight, along with a similar growth rate to that of the WT strain in *P. tricornutum*. That study also showed that *PtME* was predominantly localized to the mitochondria, although no further studies were performed on the role of ME in accumulating lipid content. The data obtained from this study along with our previously published work (Lv et al., 2016) suggested that ME1 plays an important role in NADPH supply for lipid synthesis and fatty acid desaturation in *P. tricornutum* (Wynn et al., 1999).

However, the role of ME in the provision of NADPH for lipid biosynthesis remains incompletely understood (Ratledge, 2014; Liang and Jiang, 2015). Ren et al. (2013) reported that the main source of NADPH might be from glucose-6-phosphate dehydrogenase at the early stage of fermentation, whereas ME was the main provider during the late stage in the oleaginous fungi *Schizochytrium* sp. HX-308.

Malic enzyme can promote NADPH production in oleaginous organisms; however, other some studies indicated that the enzyme cannot provide all of the NADPH required. In animal cells, although ME is important for generating NADPH for fatty acid biosynthesis (Ceddia et al., 2000), it is not the sole supplier, as 50% of the NADPH is from both glucose-6-phosphate dehydrogenase and 6-phosphogluconate dehydrogenase (Shimomura et al., 1998; Zhang et al., 2007). Pentose phosphate pathway related reactions seem to be the most likely way, in spite of a possibility that a cytosolic isocitrate dehydrogenase (ICDH) reaction with a mitochondrial ICDH function might also produce some NADPH in the reverse reaction (Ratledge, 2014).

NADPH production was not affected in the strain *Saccharomyces cerevisiae* overexpressing ME gene (lacking the mitochondria-target sequence) and exhibiting ME localization to the mitochondria due to transfer of NADPH across the mitochondrial membrane (Moreira dos Santos et al., 2004). The ME shunt proved to be diversified in this respect because of the possibility of outputting additional NADPH to the cytosol (Moreira dos Santos et al., 2004). Therefore, it is difficult to identify the correct *ME* gene involved in producing NADPH particularly for fatty acid biosynthesis (Zhang et al., 2007).

Two additional decarboxylating enzymes, which belong to the ME family, have been identified (27477 and 56501 represent NCBI predicted ME protein sequences), with both possessing mitochondria presequences in *P. tricornutum* (Kroth et al., 2008), suggesting that ME likely localizes to the mitochondria (Valenzuela et al., 2012). One of these enzymes (56501) has a dinucleotide-binding site given for NAD rather than NADP. Therefore, *P. tricornutum* appears to have two mitochondrial MEs that are either NAD- or NADP-dependent (Kroth et al., 2008). Further research is needed to determine ME1 localization.

These results along with those from other studies (Kroth et al., 2008; Xue et al., 2015) indicated that *P. tricornutum* possesses two MEs, one being an NAD cofactor (Xue et al., 2015) and the other NADP-dependent (this study).

## Conclusion

In this study, endogenous *ME1* was succeed in overexpressing in *P. tricornutum*, resulting in a significant increase in total lipid and PUFA content, and producing additional NADPH, thereby demonstrating that ME1 is NADP-dependent and plays a vital role in the supply of NADPH for lipid biosynthesis and desaturation of fatty acids in *P. tricornutum*. These findings suggested that overexpression of endogenous *ME1* represents a valid method for boosting neutral-lipid yield in diatom.

## Author Contributions

B-HZ, R-HZ, and N-NL designed the experiments, analyzed and interpreted the data, and wrote the article. Y-SW participated in algal cultivation and analyzed the data. G-PY and K-HP supervised specific experiments and gave critical revisions of the article. All authors agreed on the manuscript.

## Conflict of Interest Statement

The authors declare that the research was conducted in the absence of any commercial or financial relationships that could be construed as a potential conflict of interest.
